# Uric acid to albumin ratio is a novel predictive marker for all-cause and cardiovascular death in diabetic patients: a prospective cohort study

**DOI:** 10.3389/fendo.2024.1388731

**Published:** 2025-01-22

**Authors:** Shengnan Chen, Ming Zhang, Shouye Hu, Xiaolong Shao, Lin Liu, Zhi Yang, Kai Nan

**Affiliations:** ^1^ Department of Joint Surgery, HongHui Hospital, Xi’an Jiaotong University, Xi’an, Shaanxi, China; ^2^ Medical Department of Xi’an Jiaotong University, Xi’an, Shaanxi, China; ^3^ Department of General Practice, HongHui Hospital, Xi’an Jiaotong University, Xi’an, Shaanxi, China

**Keywords:** diabetes, all-cause death, cardiovascular death, uric acid to albumin ratio, nomogram model

## Abstract

**Background:**

Diabetes is one of the leading causes of death with an increasing prevalence worldwide. Diabetes-related premature mortality is largely preventable and reversible if identified and managed early. Accordingly, we intend to investigate the predictive value of uric acid to albumin ratio (UAR) for all-cause and cardiovascular death in diabetic patients.

**Methods:**

Univariate and multivariate Cox regression analyses were performed to identify risk factors for all-cause death of diabetic patients. The receiver operating characteristic (ROC) curves and nomogram model were used to evaluate the predictive ability of variables. Kaplan-Meier survival analysis was used to display the progression risks of diabetic patients.

**Results:**

A total of 804 diabetic patients were enrolled in the study. During the 5-year follow-up, all-cause death was found in 80 participants (9.95%) and cardiovascular death was found in 24 participants (2.99%). Age, UAR, and hsCRP were independent risk factors for all-cause death in diabetic patients after adjusting for potential confounding factors. Age and UAR had good predictive value for 1-, 3-, and 5-year all-cause death in diabetic patients, and the combination of UAR and age had the highest predictive value. An easy and intuitive prognostic nomogram model with good predictive accuracy was constructed based on age and UAR. Patients in higher quantiles of age and UAR had more rapid progression to all-cause death and higher mortality risk than patients in the lower quantiles. UAR also had good predictive value for cardiovascular death in diabetic patients.

**Conclusions:**

UAR may be a simple, cost-effective, and reliable predictive marker for all-cause and cardiovascular death in U.S. diabetic patients. The clinical utility of UAR and nomogram based on age and UAR can help physicians identify individuals at higher risk and therefore promote prevention strategies.

## 1 Introduction

Diabetes is one of the leading causes of death and disability worldwide (1). Meanwhile, the prevalence of diabetes continues to increase which poses increasing massive challenges to public health and healthcare systems worldwide (2). According to statistics, patients with diabetes have a 1.80-fold risk of all-cause death compared to people without diabetes (3). Of these, cardiovascular disease accounts for the largest proportion of deaths (4). Due to the fact that diabetes-related premature mortality is largely preventable and reversible if identified and managed early in the disease course (1), it is of great interest to search for prognostic markers with high accuracy to reduce the burden of diabetes and increase life expectancy in later life.

Correlations between uric acid and the progression of diabetes have been widely reported (5–7). However, the results of the published articles varied across studies and the exact effects of uric acid on the prognosis of diabetes remain controversial (8). Some studies support the view that higher uric acid is associated with the progression of diabetes (9, 10). A recent study showed that higher serum uric acid levels were associated with increased risks of all-cause and cardiovascular mortality in diabetes (11). In contrast, other studies showed the potentially detrimental effects of low uric acid. It has been proven that low but not high serum uric acid is associated with higher all-cause mortality, especially in those with low protein intake (12). A higher incidence of cardiovascular events and renal disease was also observed among patients with hypouricemia (13). This discrepancy may be attributable to the fact that uric acid is also a nutritional marker (14) and a powerful antioxidant (15). It has been shown that uric acid accounts for 30 to 50% of the body’s normal antioxidant capacity (5). Meanwhile, low concentrations of uric acid are considered a consequence of poor protein intake and the presence of malnutrition (8). Therefore, a single uric acid indicator does not seem to be a good predictor for the prognosis of diabetic patients.

As the most abundant circulating protein in the plasma, albumin has various physiological functions (16, 17). Serum albumin has been regarded as an indicator of nutritional status and it is also an important circulating antioxidant (17–19). Therefore, the uric acid to albumin ratio (UAR) may coordinate nutritional status and oxidative stress to better predict the prognosis of diabetic patients. To date, however, no study has been conducted to investigate the predictive value of UAR for all-cause and cardiovascular mortality among diabetic patients.

The Lancet Commission noted that it is imperative to accurately identify and characterize the populations at highest risk (1). Accordingly, we intend to investigate the predictive value of UAR in this nationally representative population-based prospective cohort study. We discovered for the first time that UAR may be a simple, cost-effective, and reliable predictive marker for physicians to identify individuals at high risk of all-cause and cardiovascular death in diabetic patients and its predictive value outperformed single uric acid. This study may provide a novel insight into improving the outcomes of diabetic patients.

## 2 Materials and methods

### 2.1 Study design

The National Health and Nutrition Examination Survey (NHANES) is a nationally representative survey designed to monitor the health of the U.S. population using a stratified, multistage probability sampling design (20). This study used data from the 2015–2016 cycle of NHANES. The mortality status of the participants was determined by the public-use National Health Interview Survey Linked Mortality Files (NHIS-LMF) through December 31, 2019. Diagnosed diabetes was defined as self-reported physician-diagnosed diabetes. The primary outcome was all-cause death. Disease-specific death was determined using the International Statistical Classification of Diseases, 10th Revision (ICD-10), and heart diseases classified by the National Center for Health Statistics (NCHS) were defined as cardiovascular death. UAR was calculated as the uric acid (μmol/L) divided by the albumin (g/L). The detailed inclusion and exclusion criteria are shown in the flow diagram (**Supplementary Figure 1**).

The protocols of NHANES have been approved by the NCHS Ethics Review Board and written informed consent was obtained from all participants.

### 2.2 Statistical analysis

Normally distributed continuous variables were presented as means and standard deviations $\left(\bar{x}\;\pm \;s\right)$ , while non-normally distributed continuous variables were presented as medians with interquartile ranges (M, IQR). Categorical variables were expressed as numbers (n). Univariate Cox regression analyses were performed to evaluate risk factors for all-cause death in diabetic patients, and the hazard ratio (HR) and 95% confidence interval (CI) were calculated. The proportional hazards (PH) assumption for the Cox proportional hazards regression was tested using the Schoenfeld residuals (21). Variables with statistical significance in univariate analysis were examined for multicollinearity. Those factors without multicollinearity were selected for multivariate Cox regression analysis (22, 23). Multicollinearity was assessed by using the variance inflation factor (VIF). VIF values greater than 10 indicated the presence of multicollinearity (24). Variables with a VIF greater than 10 were eliminated from further model construction. The time-dependent receiver operating characteristics (ROC) curve and area under the ROC curve (AUC) were used to evaluate the predictive ability of the variables (25). To calculate the 1-, 3-, and 5-year individual survival probabilities, a nomogram was constructed using prognostic variables based on the results of the multivariate analysis. The concordance index (C‐index) was calculated to estimate the discrimination of the nomogram, while the calibration curves were utilized to assess the association between the predicted and observed risk for the outcomes of the nomogram (26). Participants were classified into 4 groups based on quartiles of the variables to perform survival analysis. The Kaplan-Meier survival curve was used to display the cumulative probability of survival in diabetic patients, and the statistical comparisons were carried out using the log-rank test (27). Progression risks for each group were visualized by cumulative hazard curves (28). All analyses were performed with R (R Studio, R version 4.3.1). A two-tailed *P*-value less than 0.05 was considered statistically significant.

## 3 Results

### 3.1 Baseline characteristics

The mean age for these 804 diabetic patients was 61.18 ± 13.36 years, and females comprised 45.77% of them. The detailed baseline characteristics of enrolled participants with diabetes can be seen in **Supplementary Table 1**. During the 5-year follow-up period, all-cause death was found in 80 participants (9.95%) and cardiovascular death was found in 24 participants (2.99%).

### 3.2 Identification of potential risk factors for all-cause death in diabetic patients

Univariate Cox proportional hazards regression was performed to identify potential risk factors for all-cause death in diabetic patients, and the PH assumption was tested for each variable. And we found that gender, age, systolic blood pressure (SBP), diastolic blood pressure (DBP), body mass index (BMI), glycohemoglobin, total cholesterol (TC), low-density lipoprotein cholesterol (LDL-C), apolipoprotein B (ApoB), red blood cell (RBC), hemoglobin (Hb), platelet (PLT), high-sensitivity C-reactive protein (hsCRP), blood urea nitrogen (BUN), serum creatinine (Scr), total bilirubin (TB), lactate dehydrogenase (LDH), UAR, serum potassium, testosterone, sex hormone-binding globulin (SHBG), and marital status were associated with all-cause death in diabetic patients (**Supplementary Table 2**). All variables satisfied PH assumptions (Schoenfeld Test *P*>0.05).

### 3.3 Multivariate Cox proportional-hazards regression

Collinearity statistics showed that LDL-C, TC, and ApoB violated the assumption of collinearity (VIF values were 14.61, 11.98, and 11.00 respectively). Therefore, LDL-C, TC, and ApoB were not included in the multivariate model. Other variables that were statistically significant in the univariate analysis were then included in the multivariate Cox model. We found that age, UAR, and hsCRP were independent risk factors for all-cause death in diabetic patients after adjusting for potential confounding factors (**Table 1**).

**Table 1:** Multivariate Cox regression analysis of risk factors for all-cause death.

Variables	HR	95% CI	*P*	VIF
Gender (male *vs.* female)	2.053	0.773-5.464	0.149	
Age (year)	1.104	1.055-1.156	<0.0001	1.758
DBP (mmHg)	0.992	0.972-1.012	0.415	1.460
SBP (mmHg)	1.013	1.000-1.026	0.055	1.357
BMI (kg/m^2^)	0.998	0.949-1.050	0.938	1.463
Glycohemoglobin (%)	1.021	0.844-1.235	0.832	1.286
RBC (million/uL)	0.996	0.434-2.287	0.992	2.686
Hb (g/dL)	1.044	0.783-1.392	0.769	3.123
PLT (10^9/L)	0.996	0.991-1.001	0.113	1.289
hsCRP (mg/L)	1.029	1.008-1.051	0.007	1.339
BUN (mmol/L)	1.041	0.952-1.139	0.375	1.670
Scr (μmol/L)	1.003	0.999-1.007	0.097	1.608
TB (μmol/L)	1.024	0.964-1.088	0.444	1.277
LDH (U/L)	1.009	0.998-1.019	0.103	1.210
UAR	1.238	1.120-1.369	<0.0001	1.413
Serum potassium (mmol/L)	1.084	0.536-2.194	0.822	1.282
Testosterone (ng/dL)	1.000	0.998-1.002	0.856	1.674
SHBG (nmol/L)	1.006	0.998-1.014	0.120	1.246
Marital status				
Widowed *vs.* Married	1.441	0.690-3.011	0.331	
Divorced *vs.* Married	0.896	0.365-2.201	0.811	
Separated *vs.* Married	1.442	0.250-8.315	0.682	
Never married *vs.* Married	0.402	0.075-2.143	0.286	
Living with partner *vs.* Married	0.334	0.044-2.562	0.291	

SBP, Systolic blood pressure; DBP, Diastolic blood pressure; BMI, Body mass index; RBC, Red blood cell; Hb, Hemoglobin; PLT, Platelet; hsCRP, high-sensitivity C-reactive protein; BUN, Blood urea nitrogen; Scr, Serum creatinine; TB, Total bilirubin; LDH, Lactate dehydrogenase; UAR, Uric acid (μmol/L)/albumin(g/L); SHBG, Sex hormone-binding globulin; HR, Hazard ratio; CI, Confidence interval; VIF, Variance inflation factor.

### 3.4 Evaluation of the predictive value of prognostic factors

**Figure 1:**
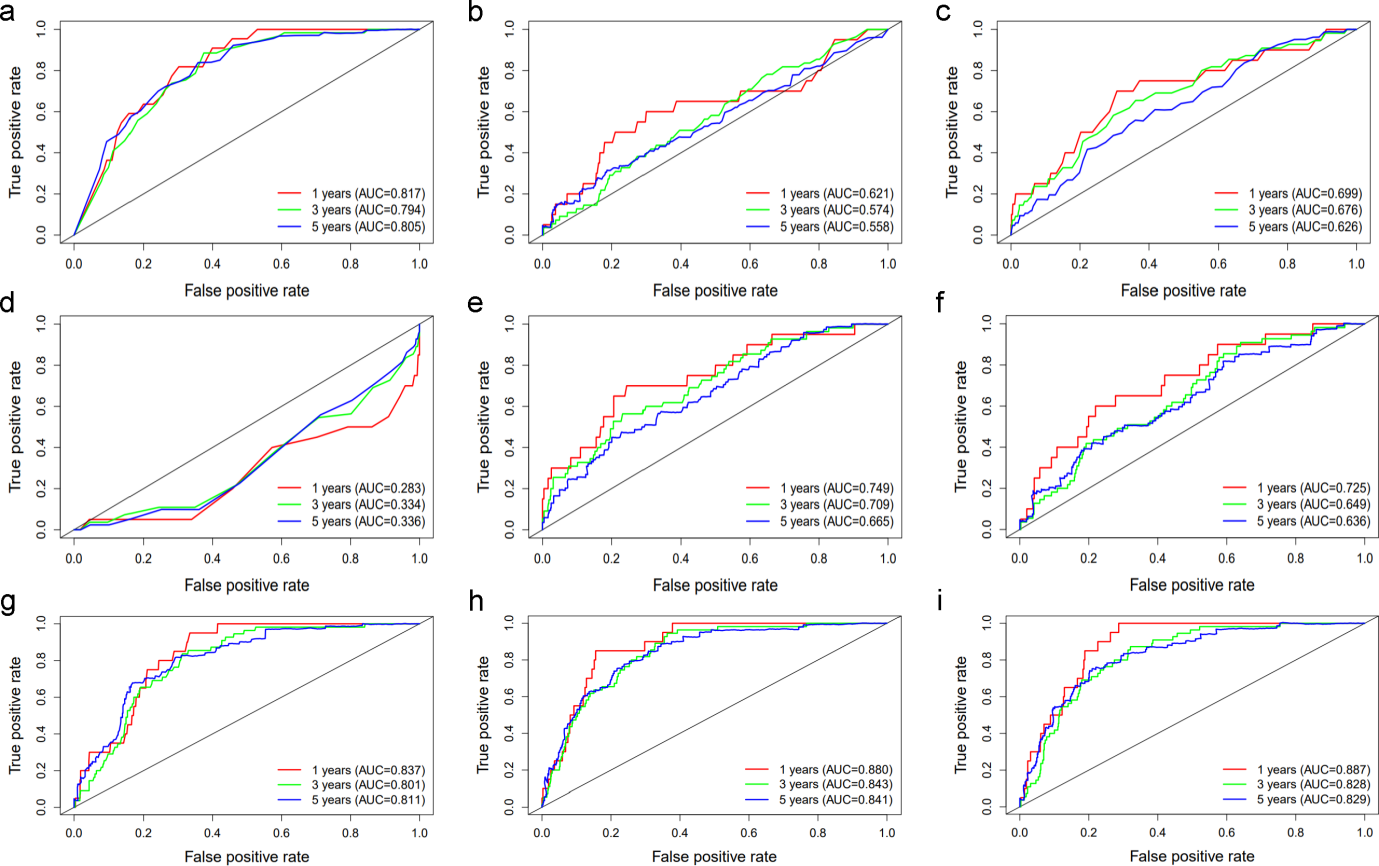
The ROC curve of **(A)** Age, **(B)** hsCRP, **(C)** Uric acid, **(D)** Albumin, **(E)** UAR, **(F)** UAR+hsCRP, **(G)** Age+hsCRP, **(H)** Age+UAR, **(I)** Age+UAR+hsCRP in predicting 1-, 3-, and 5-year all-cause death in diabetic patients The red lines represent the predictive value of the variables for 1-year all-cause mortality. The green and blue lines represent the predictive value for 3-, and 5-year all-cause mortality respectively. AUC stands for Area under the ROC Curve.

To further identify the predictive value of age, UAR, hsCRP, and their combinations, we generated time-dependent ROC curves (**Figure 1**). The results showed that age and UAR had a good predictive value for 1-, 3-, and 5-year all-cause death in diabetic patients at the univariate level compared with hsCRP, uric acid, and albumin (**Figures 1A–E**). Among the models that combined two variables, the model that combined age and UAR had the highest predictive value (**Figures 1F–H**). However, adding hsCRP as a predictor did not improve the predictive value of the combination of age and UAR (**Figure 1I**).

### 3.5 Construction and evaluation of a prognostic nomogram

**Figure 2:**
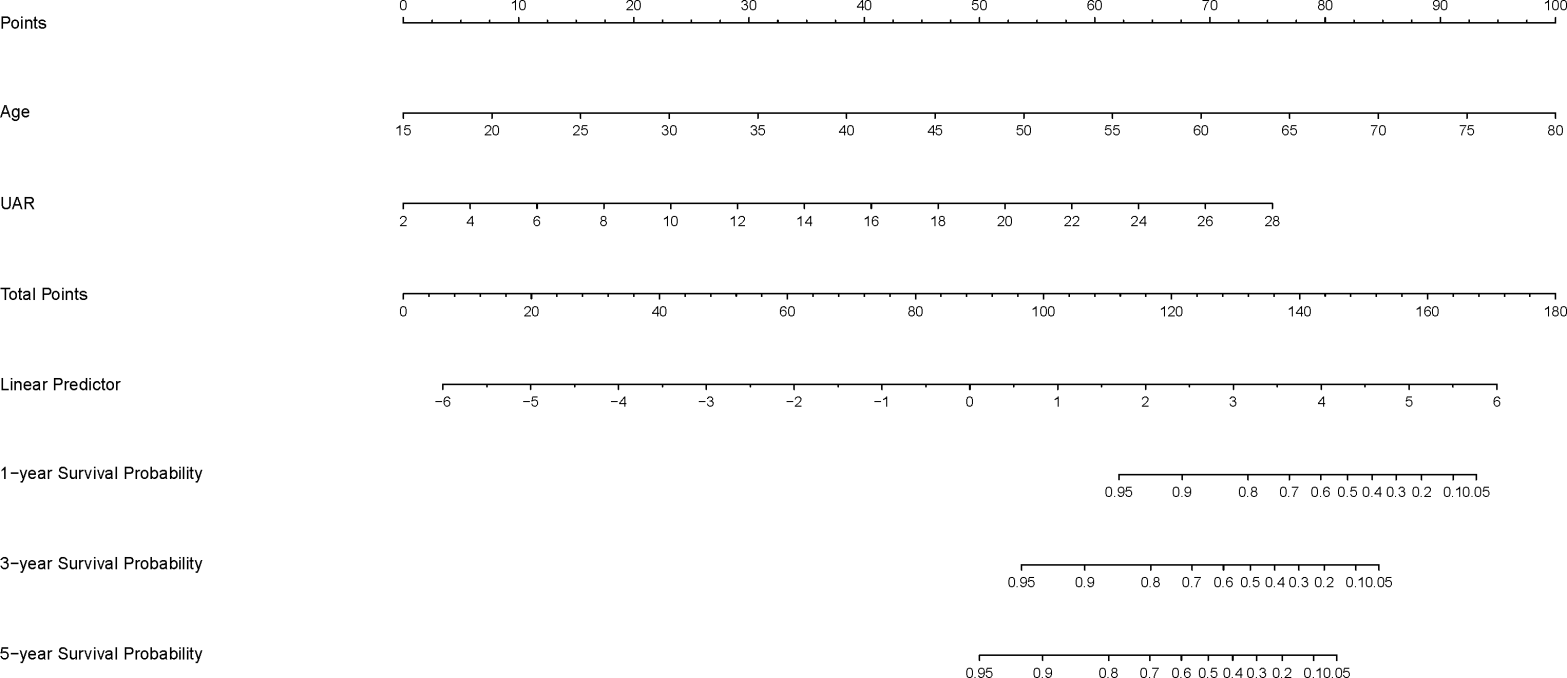
The 1-, 3-, and 5-year survival probability nomogram based on age and UAR.

**Figure 3:**
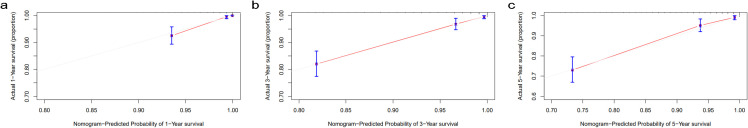
Calibration curves for **(A)** 1-year, **(B)** 3-year, and **(C)** 5-year survival probability nomogram The gray line indicates the reference line, and the red line indicates the calibration line obtained from the nomogram model.

Since the model that combined age and UAR had the best predictive value for 1-, 3-, and 5-year all-cause death in diabetic patients, we constructed a prognostic nomogram model based on age and UAR. Total points were obtained based on the predicted score calculated from the nomogram. Then, 1-, 3-, and 5-year survival probability was calculated using the nomogram’s total score axis. The prognostic nomogram based on age and UAR is shown in **Figure 2**. The C-index value for the nomogram was 0.84. The calibration curves showed that the calibration line and reference line almost entirely coincided, indicating that the nomogram model had a good predictive accuracy (**Figure 3**).

### 3.6 Survival analysis for all-cause death

**Figure 4:**
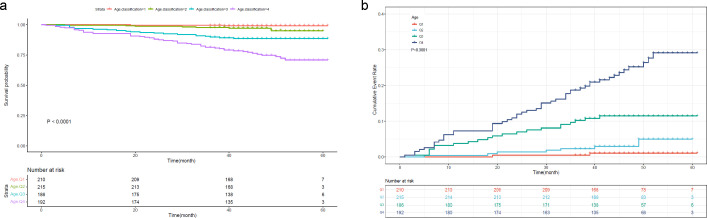
**(A)** Kaplan-Meier survival curve and **(B)** cumulative risk curve of 5-year all-cause death in diabetic patients stratified by age.

**Figure 5:**
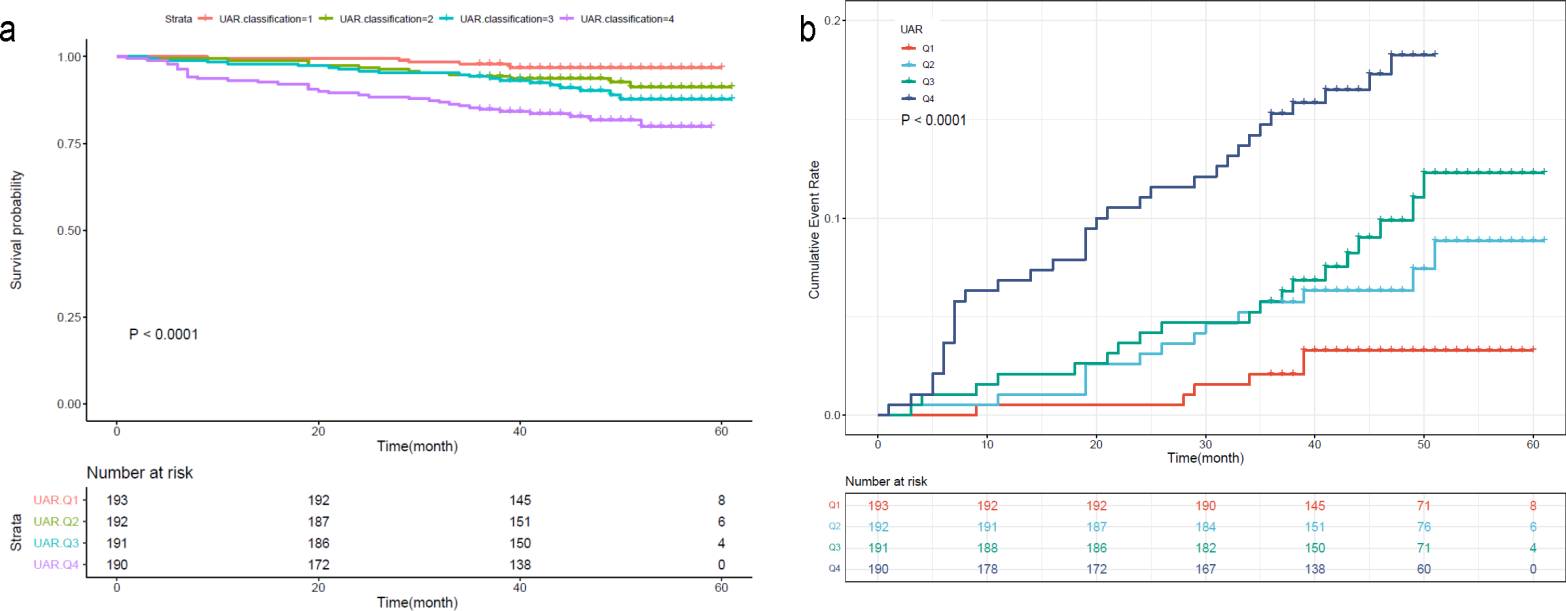
**(A)** Kaplan-Meier survival curve and **(B)** cumulative risk curve of 5-year all-cause death in diabetic patients stratified by UAR.

**Figure 6:**
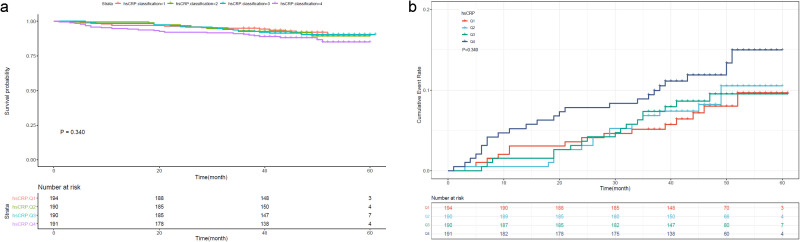
**(A)** Kaplan-Meier survival curve and **(B)** cumulative risk curve of 5-year all-cause death in diabetic patients stratified by hsCRP.

To further investigate the prognostic value of the variables, we performed survival analysis using age, UAR, and hsCRP as categorical variables. The quartile range for age was quartile 1 (Q1: 19-53), quartile 2 (Q2: 54-63), quartile 3 (Q3: 64-71), and quartile 4 (Q4: 72-80). The quartile range for UAR was quartile 1 (Q1: 2.21-6.33), quartile 2 (Q2: 6.34-7.83), quartile 3 (Q3: 7.84-9.30), and quartile 4 (Q4: 9.31-27.67). The quartile range for hsCRP was quartile 1 (Q1: 0.08-1.2), quartile 2 (Q2: 1.3-3.1), quartile 3 (Q3: 3.2-6.7), and quartile 4 (Q4: 6.8-158.1). We found that patients in higher quantiles of age had a more rapid progression to death (**Figure 4A**) and higher mortality risk (**Figure 4B**) than patients in the lower quantiles (*P*<0.0001). Similar findings were also observed in UAR group stratification (**Figure 5**). However, no significant difference was seen in the hsCRP-stratified groups (**Figure 6**, *P*=0.340).

### 3.7 Survival analysis for cardiovascular death

**Figure 7:**
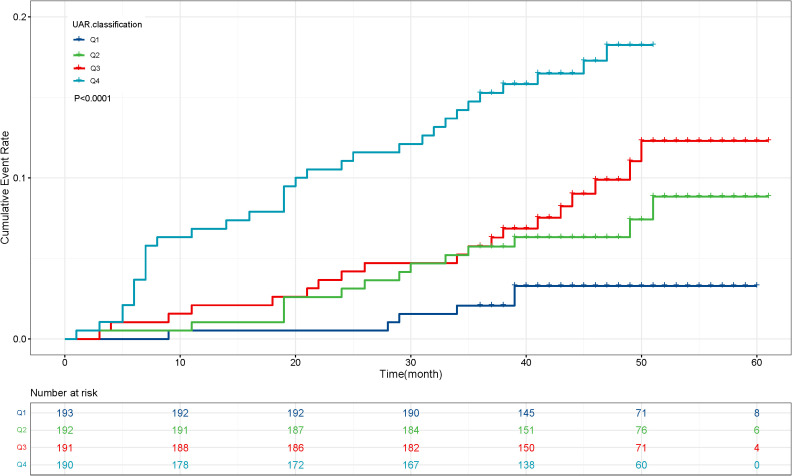
Cumulative risk curve of 5-year cardiovascular death in diabetic patients stratified by UAR.

**Figure 8:**
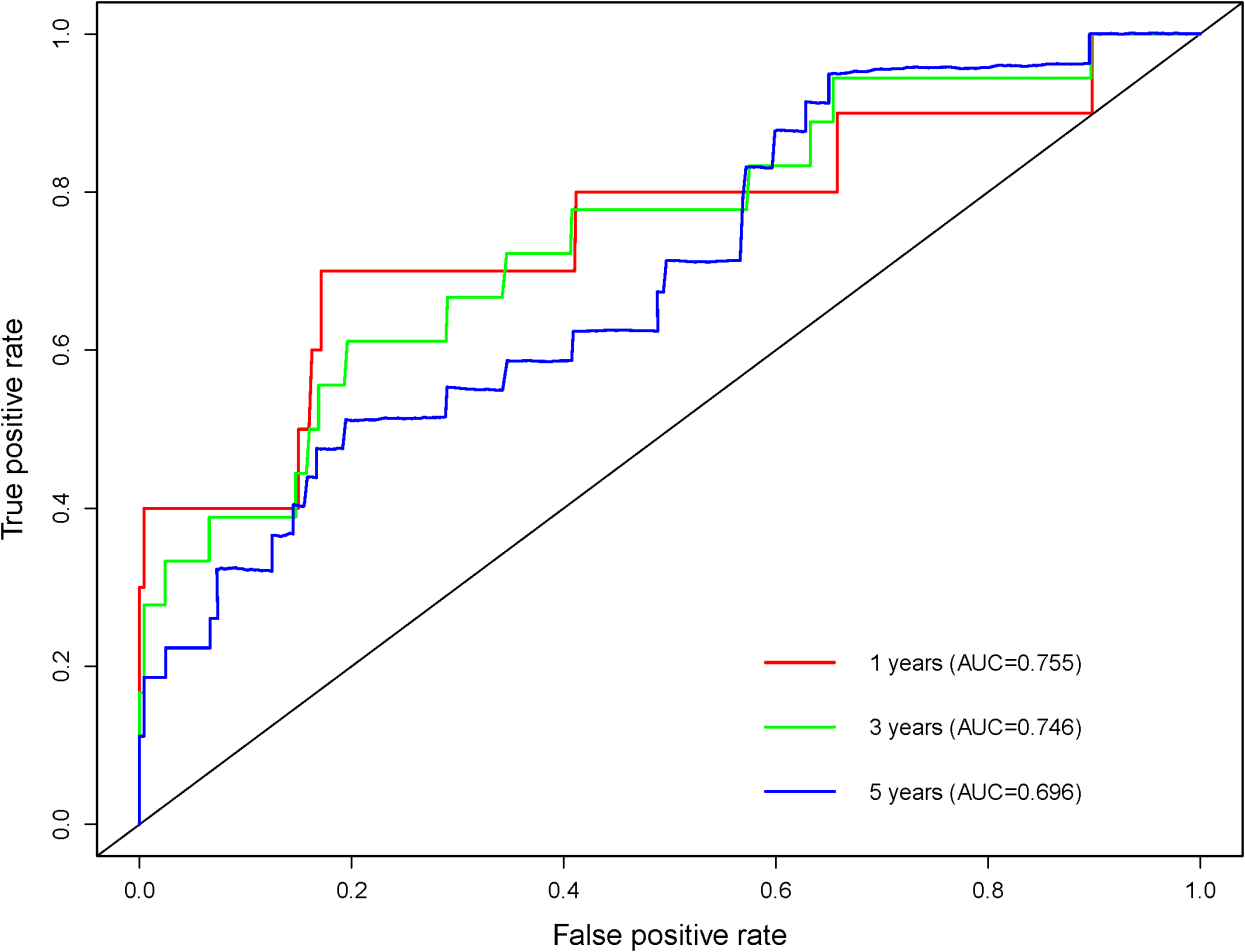
The ROC curve of UAR in predicting 1-, 3-, and 5-year cardiovascular death.

Since a large proportion of diabetic patients will die of or experience cardiovascular disease, we further analyzed the predictive value of UAR for the risk of cardiovascular death. The results showed that patients with a higher UAR quartile had a higher risk of cardiovascular death (**Figure 7**). The ROC curve showed that UAR had a good predictive value for 1-, 3-, and 5-year cardiovascular death (**Figure 8**).

## 4 Discussion

In this prospective study of a representative sample of U.S. adults, as far as we know, we discovered for the first time that UAR is a reliable predictive marker for all-cause and cardiovascular mortality in diabetic patients, and its predictive value outperformed single uric acid. The findings of the present study emphasize the importance of UAR in the risk stratification of diabetic patients, which has been underappreciated in the past. Meanwhile, we also established an easy, intuitive, and accurate nomogram to predict the 1-, 3-, and 5-year survival probability of diabetic patients. The application of this nomogram can help physicians better predict the outcome of an individual patient in clinical practice. This finding also highlights the potential benefit of joint management of hyperuricemia and nutritional status.

In the present study, age was a strong predictor of death. This may be because the mean age of the enrolled subjects was somewhat older. Although the predictive value of age was superior to UAR, age is a non-modifiable risk factor for all diseases. Available evidence suggests that even interventions commonly claimed to slow aging have little effect on most age-dependent phenotypic changes (29). Instead, UAR is a risk factor that can be modified by clinical interventions. The link between uric acid and albumin may be explained by oxidative stress and nutritional status. Traditionally, a higher serum uric acid level has been thought to be a risk factor for individuals. However, uric acid is a natural antioxidant that can scavenge reactive oxygen species, reactive nitrogen species, superoxide, hydroxyl radicals, and singlet oxygen (30). It can also reduce the consumption of other antioxidants, such as glutathione and superoxide dismutase (31). Therefore, the idea of a one-size-fits-all mentality for uric acid is outdated, as it fails to account for all the variability observed by researchers (5). Albumin is an antioxidant and anti-inflammatory protein responsible for maintaining the plasma redox state (30). Because protein-rich diets tend to contain large quantities of purines, insufficient intake of calories and protein in the control of diabetes and hyperuricemia may result in malnutrition. Therefore, integrating uric acid and albumin into a single index can better predict the prognosis of diabetic patients compared with serum uric acid or albumin alone. One possible explanation is that UAR coordinates nutritional status and oxidative stress. Since nutritional status and oxidative stress are key regulators of inflammasome activation (32), UAR may also reflect the systemic inflammation state of the body. Several other studies also demonstrated the important role of UAR. A study in a cohort of hypertensives showed that UAR is an independent predictor of high carotid intima-media thickness (33). Another study proved that UAR can be used to predict major adverse cardiac and cerebral events in aortic stenosis patients after transcatheter aortic valve implantation (34). Additionally, evidence shows that UAR is an independent predictor of new-onset atrial fibrillation in ST-elevation myocardial infarction patients (35). In the current study, we demonstrated that UAR is also a strong predictor of all-cause and cardiovascular death in diabetic patients.

It is worth mentioning that UAR is a simple and cost-effective method because uric acid and albumin were routinely tested in diabetic patients. Our proposed nomogram based on UAR and age is simple and practical with high accuracy. With this method, physicians can identify patients at high risk of all-cause and cardiovascular mortality and thus take early interventions to improve the prognosis of diabetic patients.

The strengths of this study are as follows. First, to the best of our knowledge, this is the first study to investigate the predictive value of UAR in predicting all-cause and cardiovascular death in diabetic patients. Second, this is a prospective cohort study in a nationally representative sample which gives a more reliable result. Third, we adjusted as many confounding factors as possible and adopted various predictive methods to reach a convincing conclusion. Last but not least, we established an easy, intuitive, and accurate nomogram to predict the 1-, 3-, and 5-year survival probability of diabetic patients. The application of this nomogram can help physicians better predict the outcome of an individual patient in clinical practice.

The limitations of this study are also worth mentioning. First, the questionnaire did not classify the types of diabetes. Second, the follow-up period was only five years; we were not able to assess the long-term predictive value of UAR. Third, despite our effort to adjust for risk factors, residual unmeasured confounders may exist. Lastly, while the present findings provide valuable insights into the characteristics and outcomes of diabetic patients in the U.S. population, the generalizability of these results to other populations may be limited. Hence, multinational cohorts with a longer follow-up period are needed to further verify these findings.

## 5 Conclusions

We discovered for the first time that UAR may be a simple, cost-effective, and reliable predictive marker for all-cause and cardiovascular death in diabetic patients and its predictive value outperformed single uric acid. Our proposed nomogram based on UAR and age is simple, intuitive, and practical with high accuracy. The clinical utility of UAR and nomogram based on age and UAR can help physicians identify individuals at higher risk and therefore promote prevention strategies. This study provides a novel insight into improving the outcomes of diabetic patients. This finding also highlights the potential benefit of joint management of hyperuricemia and nutritional status.

## Data Availability

The original contributions presented in the study are included in the article/**Supplementary Material**. Further inquiries can be directed to the corresponding author/s.

## Supplementary material

Supplementary Figure 1 Flow diagram of inclusion and exclusion criteria.
