# Supplementary figures and images for: Uric acid to albumin ratio is a novel predictive marker for all-cause and cardiovascular death in diabetic patients: a prospective cohort study
Source: Front Endocrinol (Lausanne). 2025 Jan 22;15:1388731. doi: 10.3389/fendo.2024.1388731 (PMC11794066; doi:10.3389/fendo.2024.1388731)

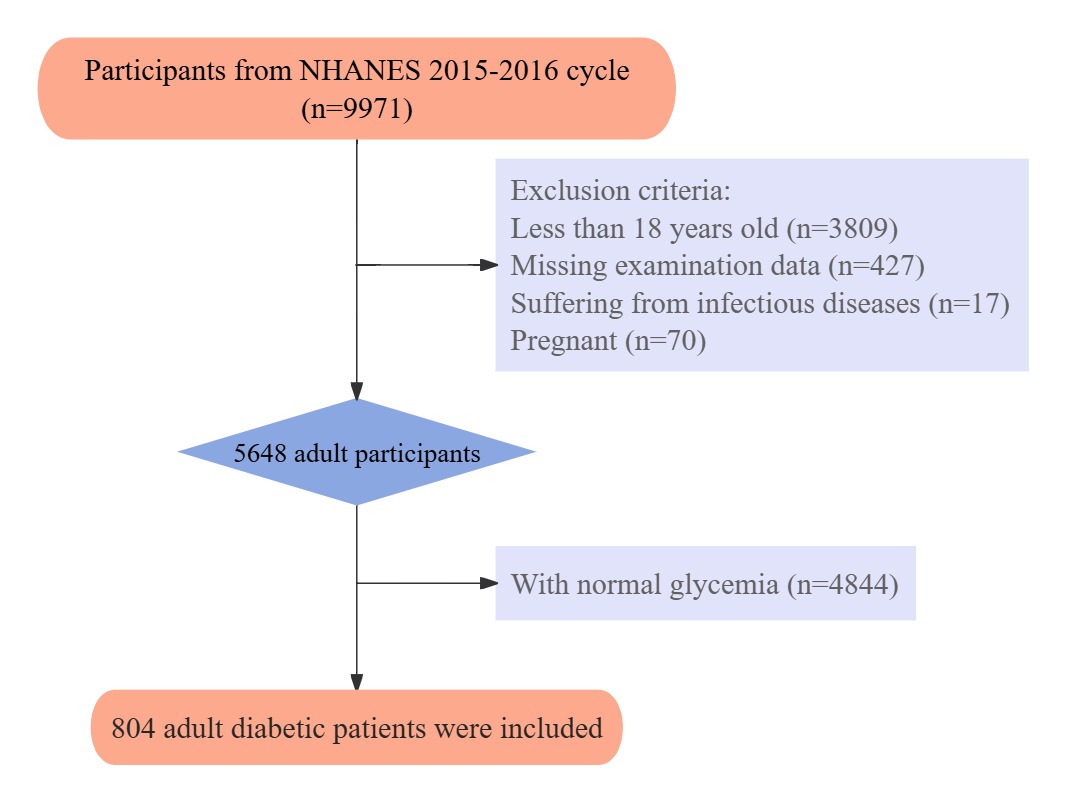

Supplement: Supplementary Figure 1 — Flow diagram of inclusion and exclusion criteria. [file Image1.jpeg]
