# Supplementary material for: Uric acid to albumin ratio is a novel predictive marker for all-cause and cardiovascular death in diabetic patients: a prospective cohort study
Source: Front Endocrinol (Lausanne). 2025 Jan 22;15:1388731. doi: 10.3389/fendo.2024.1388731 (PMC11794066; doi:10.3389/fendo.2024.1388731)
Supplement: Supplementary file 2 [file Table1.docx]

Supplementary Table 1. Demographic and clinical baseline characteristics of enrolled participants with diabetes

| Variables | Participants with diabetes |
| --- | --- |
| Gender (male/female, n) | 436/368 |
| Age (year, $\bar{x}$±s) | 61.18±13.36 |
| SBP (mmHg, $\bar{x}$±s) | 132.54±19.65 |
| DBP (mmHg, $\bar{x}$±s) | 77.97±13.74 |
| Pulse (beats/min, $\bar{x}$±s) | 73.66±12.23 |
| BMI (kg/m^2^, (M, IQR)) | 31.50, 8.80 |
| FPG (mmol/L, (M, IQR)) | 8.27, 4.53 |
| OGTT 2h glucose (mmol/L, (M, IQR)) | 8.69, 6.00 |
| Serum insulin (pmol/L, (M, IQR)) | 70.62, 86.16 |
| Glycohemoglobin (%, (M, IQR)) | 7.57, 2.10 |
| TG (mmol/L, (M, IQR)) | 1.03, 0.57 |
| TC (mmol/L, $\bar{x}$±s) | 4.60±1.15 |
| LDL-C (mmol/L, (M, IQR)) | 2.48, 1.32 |
| HDL-C (mmol/L, (M, IQR)) | 1.19, 0.49 |
| ApoB (g/L, (M, IQR)) | 0.87, 0.37 |
| RBC (million/uL, $\bar{x}$±s) | 4.69±0.52 |
| Hb (g/dL, $\bar{x}$±s) | 13.76±1.64 |
| WBC (10^9/L, $\bar{x}$±s) | 7.39±1.93 |
| PLT (10^9/L, $\bar{x}$±s) | 234.17±69.87 |
| hsCRP (mg/L, (M, IQR)) | 3.10, 5.50 |
| BUN (mmol/L, (M, IQR)) | 5.36, 2.41 |
| Scr (μmol/L, (M, IQR)) | 73.37, 30.94 |
| TB (μmol/L, (M, IQR)) | 8.55, 5.13 |
| LDH (U/L, $\bar{x}$±s) | 129.51±27.99 |
| UAR (uric acid/albumin, (M, IQR)) | 7.84, 2.96 |
| Serum potassium (mmol/L, $\bar{x}$±s) | 4.08±0.38 |
| Serum calcium (mmol/L, $\bar{x}$±s) | 2.35±0.09 |
| Serum sodium (mmol/L, $\bar{x}$±s) | 139.62±2.10 |
| Serum phosphorus (mmol/L, (M, IQR)) | 1.16, 0.27 |
| Estrogens (pg/mL, (M, IQR)) | 18.70, 26.85 |
| Testosterone (ng/dL, (M, IQR)) | 140.00, 316.15 |
| SHBG (nmol/L, (M, IQR)) | 43.61, 33.27 |
| Hypertension (Yes/No, n) | 558/246 |
| Use of antihypertensive drugs (Yes/No, n) | 487/317 |
| Use of oral hypoglycemic agents (Yes/No, n) | 554/250 |
| Use of insulin (Yes/No, n) | 236/568 |
| Use of aspirin (Yes/No, n) | 359/445 |
| Use of urate-lowering drugs (Yes/No, n) | 81/723 |
| Marital status (Never married/ Living with partner/Married/ Separated/Divorced/ Widowed, n) | 79/53/451/28/102/91 |
|  |  |
| Education level (Less than 9th grade/9-11th grade/High school graduate/Some college or AA/ College graduate or above, n) | 151/113/176/222/142 |
|  |  |
| Recent tobacco use (Yes/No/unknown, n) | 5/182/617 |
| Drinking frequency (Day, (M, IQR)) | 0.04, 0.34 |

Abbreviations: $\bar{x}$±s: Mean and standard deviation, M: Median, IQR: Interquartile range, n: Number, SBP: Systolic blood pressure, DBP: Diastolic blood pressure, BMI: Body mass index, FPG: Fasting plasma glucose, OGTT: Oral glucose tolerance test, TC: Total cholesterol, LDL-C: Low-density lipoprotein cholesterol, HDL-C: High-density lipoprotein cholesterol, ApoB: Apolipoprotein B, RBC: Red blood cell, Hb: Hemoglobin, WBC: White blood cell, PLT: Platelet, hsCRP: high-sensitivity C-reactive protein, BUN: Blood urea nitrogen, Scr: Serum creatinine, TB: Total bilirubin, LDH: Lactate dehydrogenase, UAR: Uric acid (umol/L)/albumin(g/L), SHBG: Sex hormone-binding globulin.
