# Supplementary material for: Uric acid to albumin ratio is a novel predictive marker for all-cause and cardiovascular death in diabetic patients: a prospective cohort study
Source: Front Endocrinol (Lausanne). 2025 Jan 22;15:1388731. doi: 10.3389/fendo.2024.1388731 (PMC11794066; doi:10.3389/fendo.2024.1388731)
Supplement: Supplementary file 3 [file Table2.docx]

Supplementary Table 2. Univariate Cox regression analysis for all-cause death in diabetic patients

| Variables | *P* value | HR (95% CI) | Schoenfeld Test *P* |
| --- | --- | --- | --- |
| Gender (male *vs.* female) | 0.002 | 2.179 (1.342-3.534) | 0.856 |
| Age (year) | <0.0001 | 1.117 (1.086-1.149) | 0.592 |
| SBP (mmHg) | 0.008 | 1.014 (1.004-1.024) | 0.355 |
| DBP (mmHg) | <0.0001 | 0.969 (0.957-0.982) | 0.575 |
| Pulse (beats/min) | 0.133 | 0.986 (0.968-1.004) | 0.492 |
| BMI (kg/m^2^) | 0.031 | 0.963 (0.931-0.997) | 0.787 |
| FPG (mmol/L) | 0.166 | 0.933 (0.845-1.029) | 0.448 |
| OGTT 2h glucose (mmol/L) | 0.257 | 0.711 (0.394-1.282) | 0.190 |
| Serum insulin (pmol/L) | 0.878 | 1.000 (0.998-1.001) | 0.358 |
| Glycohemoglobin (%) | 0.028 | 0.849 (0.733-0.983) | 0.064 |
| TG (mmol/L) | 0.362 | 0.798 (0.491-1.296) | 0.280 |
| TC (mmol/L) | <0.0001 | 0.562 (0.438-0.722) | 0.248 |
| LDL-C (mmol/L) | 0.004 | 0.460 (0.272-0.777) | 0.171 |
| HDL-C (mmol/L) | 0.864 | 1.049 (0.609-1.805) | 0.359 |
| ApoB (g/L) | 0.003 | 0.064 (0.011-0.381) | 0.521 |
| RBC (million/uL) | <0.0001 | 0.415 (0.274-0.630) | 0.665 |
| Hb (g/dL) | 0.002 | 0.805 (0.703-0.922) | 0.848 |
| WBC (10^9/L) | 0.861 | 0.991 (0.894-1.098) | 0.935 |
| PLT (10^9/L) | <0.0001 | 0.991 (0.987-0.995) | 0.811 |
| hsCRP (mg/L) | <0.0001 | 1.018 (1.008-1.027) | 0.716 |
| BUN (mmol/L) | <0.0001 | 1.179 (1.134-1.226) | 0.807 |
| Scr (μmol/L) | <0.0001 | 1.004 (1.002-1.005) | 0.928 |
| TB (μmol/L) | 0.004 | 1.056 (1.018-1.095) | 0.483 |
| LDH (U/L) | 0.001 | 1.012 (1.005-1.020) | 0.167 |
| UAR (uric acid/albumin) | <0.0001 | 1.270 (1.194-1.350) | 0.167 |
| Serum potassium (mmol/L) | <0.0001 | 2.859 (1.666-4.908) | 0.398 |
| Serum calcium (mmol/L) | 0.075 | 0.099 (0.008-1.262) | 0.087 |
| Serum sodium (mmol/L) | 0.482 | 1.033 (0.944-1.129) | 0.066 |
| Serum phosphorus (mmol/L) | 0.890 | 1.091 (0.319-3.734) | 0.133 |
| Estrogens (pg/mL) | 0.207 | 0.993 (0.982-1.004) | 0.742 |
| Testosterone (ng/dL) | 0.041 | 1.001 (1.000-1.002) | 0.855 |
| SHBG (nmol/L) | <0.0001 | 1.008 (1.004-1.012) | 0.281 |
| Hypertension (No *vs.* Yes) | 0.202 | 0.715 (0.427-1.197) | 0.979 |
| Use of antihypertensive drugs  (No *vs.* Yes) | 0.826 | 0.902 (0.361-2.252) | 0.071 |
| Use of oral hypoglycemic agents (No *vs.* Yes) | 0.147 | 1.400 (0.889-2.205) | 0.357 |
| Use of insulin (No *vs.* Yes) | 0.168 | 0.725 (0.460-1.144) | 0.153 |
| Use of aspirin (No *vs.* Yes) | 0.244 | 0.637 (0.298-1.360) | 0.100 |
| Use of urate-lowering drugs (No vs. Yes) | 0.455 | 1.373 (0.597-3.154) | 0.435 |
| Marital status |  |  | 0.771 |
| Widowed *vs.* Married | 0.002 | 2.393 (1.389-4.123) |  |
| Divorced *vs.* Married | 0.633 | 1.176 (0.605-2.288) |  |
| Separated *vs.* Married | 0.341 | 1.646 (0.590-4.596) |  |
| Never married *vs.* Married | 0.151 | 0.423 (0.131-1.367) |  |
| Living with partner *vs.* Married | 0.232 | 0.421 (0.102-1.741) |  |
| Education level |  |  | 0.782 |
| 9-11th grade *vs.* less than 9th grade | 0.298 | 1.472 (0.711-3.050) |  |
| High school graduate/GED *vs.* less than 9th grade | 0.967 | 0.985 (0.481-2.018) |  |
| Some college or AA *vs.* less than 9th grade | 0.582 | 1.204 (0.623-2.327) |  |
| Recent tobacco use (Yes *vs.* No) | 0.998 | 0(0-Inf) | 1.000 |
| Drinking frequency (Day) | 0.076 | 0.129 (0.013-1.243) | 0.052 |

Abbreviations: SBP: Systolic blood pressure, DBP: Diastolic blood pressure, BMI: Body mass index, FPG: Fasting plasma glucose, OGTT: Oral glucose tolerance test, TC: Total cholesterol, LDL-C: Low-density lipoprotein cholesterol, HDL-C: High-density lipoprotein cholesterol, ApoB: Apolipoprotein B, RBC: Red blood cell, Hb: Hemoglobin, WBC: White blood cell, PLT: Platelet, hsCRP: high-sensitivity C-reactive protein, BUN: Blood urea nitrogen, Scr: Serum creatinine, TB: Total bilirubin, LDH: Lactate dehydrogenase, UAR: Uric acid (umol/L)/albumin(g/L), SHBG: Sex hormone-binding globulin, HR: Hazard ratio, CI: Confidence interval.
